# Periodontal Microbial Profiles Across Periodontal Conditions in Pediatric Subjects: A Narrative Review

**DOI:** 10.3390/microorganisms13081813

**Published:** 2025-08-03

**Authors:** Federica Di Spirito, Maria Pia Di Palo, Giuseppina De Benedetto, Federica Piedepalumbo, Marzio Galdi, Davide Cannatà, Noemi Cafà, Maria Contaldo

**Affiliations:** 1 Department of Medicine, Surgery and Dentistry, University of Salerno, via S. Allende, 84081 Baronissi, Italy; mariapia140497@gmail.com (M.P.D.P.);; 2 Multidisciplinary Department of Medical-Surgical and Odontostomatological Specialties, University of Campania “Luigi Vanvitelli”, 80138 Naples, Italy; maria.contaldo@unicampania.it

**Keywords:** periodontitis, gingivitis, periodontal diseases, dental plaque, oral microbiota, bacteria, viruses, fungi, oral dysbiosis, children

## Abstract

Periodontal diseases in pediatric subjects represent a challenging and relatively underexplored area compared to the extensive data available about periodontal diseases in adults. The present narrative review aims to explore the periodontal status and the related subgingival and/or salivary microbial profiles in pediatric subjects (≤18 years), focusing also on the state of health or systemic diseases. In healthy periodontium, early colonizers, such as *Streptococcus* and *Actinomyces* spp., dominate the subgingival microbiota, supporting an eubiosis state. Low levels of *Candida albicans* and latent Herpesviridae may be detected. In gingivitis, the microbial profile shifts towards more pathogenic species, including *Prevotella intermedia* and *Fusobacterium nucleatum*. In necrotizing gingivitis, typically affecting systemically compromised children, the microbial profile is characterized by spirochetes, *Fusobacterium*, and *Prevotella intermedia*. Viral coinfections—especially with HSV, CMV, and EBV—are more frequently detected. In periodontitis, the microbiota was dominated by red complex pathogens along with *Aggregatibacter actinomycetemcomitans* in the aggressive forms, especially in systemically compromised children, as Herpesviridae reactivation and co-infections. Fungal involvement is less well characterized; *Candida albicans* may be present, particularly in cases of severe immune suppression. Nevertheless, the lack of pediatric longitudinal studies investigating periodontal disease progression after periodontal treatment and related changes in microbiological composition limited the understanding and exploration of the oral microbiota over time.

## 1. Introduction

Periodontal diseases in pediatric (≤18 years of age) subjects represent a challenging and relatively underexplored area compared to the extensive data available about periodontal diseases in adults.

The classification of periodontal diseases in pediatric subjects has evolved over the past decades. Initially, the American Academy of Periodontology (1977) [1] identified “juvenile periodontitis” and “chronic marginal periodontitis” as distinct categories. By 1986, juvenile periodontitis was subdivided into prepubertal, localized, and generalized forms [1]. The term “early-onset periodontitis” was introduced in 1989, categorizing various forms based on age and pattern criteria: prepubertal and juvenile periodontitis (both localized and generalized), and the rapidly progressive periodontitis [2]. Localized juvenile periodontitis typically involves incisors and first molars through early adulthood (about 20 years), while generalized forms affect multiple teeth by the second and third decades of life [2]. The International Workshop for Periodontal Disease and Conditions introduced the term “aggressive periodontitis,” eliminating the previous age-based distinctions. In 2017, the term “periodontitis” unified both chronic and aggressive periodontitis [3].

As the terminology used in the literature varies according to the period of publication, current knowledge on periodontal disease in pediatric subjects is fragmented and heterogeneous.

According to the 2017 World Workshop, periodontitis is a microbially-associated, host-mediated, oral inflammatory disease—resulting in a dynamic interplay among microorganisms, host responses, and environment—that progressively destroys the supporting tooth, up to loss of the tooth itself [3].

With respect to periodontitis, gingivitis is a reversible periodontal disease that is more common among children and is characterized by gingival inflammation triggered by plaque, which is accompanied by redness, bleeding, and swelling, without the destruction of periodontal tissue supports [3,4,5].

Necrotizing gingivitis and periodontitis are rapidly progressing inflammatory periodontal diseases characterized by interdental papillae necrosis, pain, and spontaneous bleeding [3,6,7]. It is typically observed in poor oral hygiene conditions, or in immunocompromised or malnourished subjects [3,6,7].

Even if the destructive forms of periodontal diseases are less common in systemically healthy pediatric subjects, they can manifest more frequently and in a more severe form in pediatric subjects affected by systemic conditions, such as genetic syndromes (e.g., Down syndrome or Papillon–Lefèvre syndrome) [8,9], endocrine and metabolic diseases (e.g., uncontrolled diabetes) [8,10], or congenital and acquired immunodeficiencies [8,11].

Concerning the pathogenesis of periodontal diseases, recent evidence suggests that beyond well-established periodontal bacterial pathogens—*Tannarella forsythia*, *Treponema denticola*, and *Porphyromonas gingivalis*—viruses may also contribute to disease progression. This hypothesis arises from clinical observations in pediatric patients, in which some forms of periodontitis showed rapid periodontal tissue progression and destruction despite low biofilm accumulation [12,13].

In particular, some evidence highlighted the presence of human herpesviruses (HHVs) at periodontal sites, which can affect the immune defense of cells infected by HHVs, thereby favoring the virulence of periodontal pathogenic bacteria [14,15]. Exploring the presence and role of HHVs in the periodontal sites of pediatric subjects could be particularly relevant, as latent HHV reactivation during puberty may be triggered by hormonal imbalances, pharmacologic agents, or other external stressors [14].

In this complex and dynamic scenario, fungi may also play a role in the pathogenesis of periodontal diseases, particularly *Candida albicans*, which appears to interfere with microbial mechanisms of host defense evasion [15].

Furthermore, interactions and synergistic effects among bacteria, viruses, and fungi may be amplified by systemic conditions that increase periodontal disease susceptibility and disrupt the oral microbiome, potentially predisposing pediatric patients with systemic disorders to more severe periodontal manifestations [8,11,16]. Conversely, periodontal diseases themselves have been associated with systemic health issues, including cardiovascular disease, hypertension, and certain cancers, and are often exacerbated in conditions like rheumatoid arthritis and other autoimmune disorders, due to altered immune responses [17]. This highlights the bidirectional relationship between periodontal and systemic health [17].

Although periodontal pathogenic bacteria, HHVs, and fungi have been more extensively studied in adult periodontitis, current evidence regarding their role in pediatric periodontal diseases remains limited and fragmented [18].

Therefore, the present narrative review aimed to explore the periodontal status (ranging from healthy periodontium to gingivitis, periodontitis, and necrotizing gingivitis/periodontitis) and the related subgingival and/or salivary microbial profiles of pediatric subjects (≤18 years of age), focusing also on the state of health or systemic diseases affecting the pediatric population.

## 2. Materials and Methods

**Figure 1:**
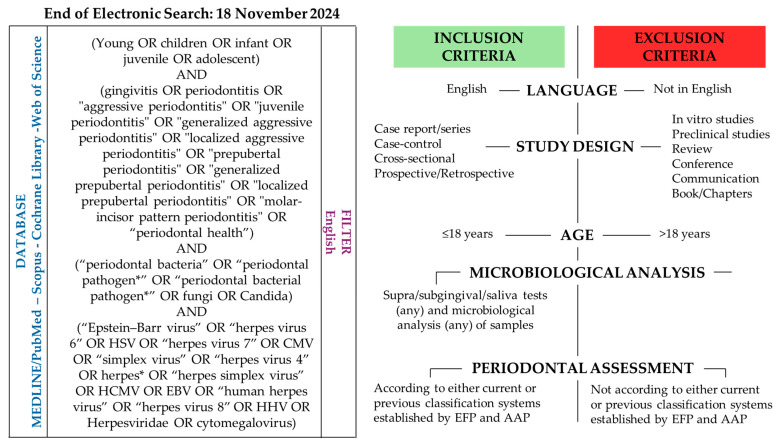
Electronic search strategy and the eligibility criteria [3].

The electronic search was conducted by two independent reviewers (F.D.S. and G.B.D.) to retrieve relevant research about the subgingival and/or salivary microbial profiles of pediatric subjects (≤18 years of age) and the related periodontal status. The electronic search strategy and the eligible criteria are presented in Figure 1.

A manual search of the reference lists of records retrieved through the electronic search was also performed to ensure a comprehensive literature search strategy and to minimize the risk of overlooking seminal studies.

Inclusion criteria were case reports, case series, case–control, cross-sectional, and prospective/retrospective studies in the English language, without restrictions on year of publication, sample size, or children’s gender. Participants were pediatric subjects (≤18 years) who underwent supra/subgingival/saliva tests (any) and microbiological analysis (any) of samples. Periodontal conditions had to be diagnosed according to either current or previous classification systems established by the EFP and AAP [3].

Exclusion criteria were studies not in English, in vitro/preclinical in vivo studies, reviews, conferences, oral communications, and books/chapters; adult subjects (>18 years); studies that did not evaluate the microbiological content of supra/subgingival/saliva tests; and subjects with periodontal conditions not diagnosed according to either current or previous classification systems established by the EFP and AAP [3].

All relevant and eligible references were collected and managed by the two independent reviewers (F.D.S. and G.D.B.) using Mendeley Reference Manager software, version 2.135.0. A third independent reviewer (M.C.) was consulted when the two reviewers disagreed in order to resolve doubts through discussion.

In the present narrative review, data from relevant collected studies were qualitatively synthesized.

To facilitate clarity and thematic progression, data were organized following a dual approach: first, by introducing the general background on microbial sampling methods and microbial taxa commonly found in pediatric periodontal sites; then, by presenting specific microbial profiles (in order: bacterial, viral, and fungal profiles) across periodontal conditions according to increasing clinical severity, ranging from healthy periodontium to gingivitis, necrotizing forms, and periodontitis. A final section summarized the microbial findings retrieved across all periodontal conditions in pediatric subjects with systemic conditions in relation to the specific systemic diseases.

## 3. Microbiological Sampling and Analysis of Periodontal Microbiota in Pediatric Subjects

Reliable sampling is essential to detecting the microbial profiles in both oral health and disease conditions, maintaining their integrity [19]. Following the sample collection, rigorous statistical analysis capable of detecting microbial associations and patterns is essential to interpret the extensive datasets generated by advanced microbiological techniques [20]. A critical component of microbiological findings interpretation involves correlating microbial results with clinical parameters [20], such as periodontal probing depth (PPD), clinical attachment level (CAL), and radiographic bone level, in periodontal disease research. Understanding the correlations between periodontal status, periodontal microbial profile, and general health can lead to the development of targeted treatments to restore a healthy microbial profile.

Despite recent advances, microbiological analysis still has several limitations, such as variability in sampling methods, the impact of individual host factors on microbial profile, and the risk of contamination during laboratory procedures [21,22].

### 3.1. Periodontal Microbiological Sampling

Periodontal microbiological sampling used in pediatric subjects with periodontal diseases employs various approaches, ranging from swabbing, scraping, and the application of sterile paper points [12,23,24,25,26,27]. The insertion of sterile paper points into periodontal pockets to absorb crevicular fluid and related microorganisms is the most commonly used periodontal microbiological sampling method. This technique has the advantage of accessing narrow or deep periodontal pockets, compared to swabbing or scraping methods [28].

The proper handling and transportation of collected samples are essential to preventing contamination and degradation, which could compromise the accuracy of the results [29]. Samples must be obtained using sterile techniques and placed into appropriate transport media, such as anaerobic transport medium or phosphate-buffered saline, to maintain microbial viability. For sample transport, it is recommended to maintain a temperature between 2 and 8 °C and deliver samples to the laboratory as soon as possible within 24 h [29]. Finally, ethical aspects of microbiological sampling in pediatric subjects require particular attention due to the adherence to institutional and international ethical standards needed, as well as obtaining informed consent from the children’s legal guardians and assent from the child when appropriate. Additionally, the study protocol must be approved by a local ethics committee before the microbiological sampling [30].

### 3.2. Periodontal Microbiological Analysis

Microbiological methods used to analyze the bacterial, viral, and fungal profiles in pediatric patients with periodontal diseases have encompassed a range of approaches, from traditional culture-dependent techniques to advanced molecular methodologies [12,23,24,25,26,27].

Culture-dependent techniques require propagation on selective growth media (as liquid nutrient broths or on solid agar surfaces) under laboratory-controlled conditions [31]. These culture-dependent techniques are valuable for identifying specific bacteria, especially if present in low abundance. However, their major limitation is the inability to allow the growth of all bacteria, thereby leading to an underestimation of the overall microbial sample diversity [31]. This issue is particularly relevant in complex microbial environments, such as the oral microbiome [32].

To overcome these limitations, advanced molecular techniques that are capable of sequencing DNA have been extensively adopted, including next-generation sequencing (NGS) and polymerase chain reaction (PCR) [22]. PCR selectively amplifies target DNA sequences, enabling the identification of specific bacterial species [29,33]. NGS offers a comprehensive characterization of the sample’s microbial profile, characterizing both species diversity and related abundance [22]. Due to its high sensitivity and specificity, quantitative PCR is widely employed to quantify the concentration of target bacterial DNA within a sample, thereby enabling the assessment of bacterial load and recording changes in microorganism abundance over time [34].

Metagenomics enables the direct sequencing of genetic material without prior culturing, thereby capturing a broader spectrum of microbial taxa, including non-culturable organisms. Studies employing metagenomic approaches have demonstrated that culture-dependent techniques often detect less than 1% of a sample’s bacterial diversity [35]. Furthermore, the interpretation of metagenomic data is facilitated by bioinformatics tools [36].

## 4. Periodontal Microbiota in Pediatric Subjects

The oral microbiome is a balanced, dynamic, and symbiotic community comprising bacteria, viruses, fungi, protozoa, and archaea that establishes a homeostatic relationship with the host [37,38]. Although bacterial species constitute the majority of the oral microbiome, other microbial communities—such as the virobiome and mycobiome—also play important roles in maintaining a healthy status. The dynamic interaction between the oral microbiome and the host may influence the oral microbiome profile, potentially driving a shift from a well-balanced “eubiotic” state to a not-balanced “dysbiotic” state, thereby contributing to the development of gingivitis and periodontitis, particularly under conditions of local inflammation or impaired host defenses [37,39].

In line with the concepts of inter-kingdom and polymicrobial synergy, as well as to the dysbiosis theory, periodontal disease is not attributed to a single pathogen but rather to a synergistic and coordinated microbial community capable of driving periodontal tissue destruction [39,40].

Therefore, characterizing the periodontal microbial profile—including bacterial, viral, and fungal components—in both systemically healthy and medically compromised pediatric subjects, across periodontal health and disease states, is essential for a comprehensive understanding of oral microbial dynamics.

### 4.1. Periodontal Bacteria in Pediatric Subjects

Socransky classified the predominant periodontal bacterial pathogens in the “Socransky complexes” using a color-coded system that groups bacteria according to their association with periodontal health or disease and shared pathogenic characteristics [41]. The key microbiological characteristics and role in periodontal health and diseases of the predominant periodontal bacterial pathogens are summarized in Table 1.

### 4.2. Periodontal Viruses in Pediatric Subjects

Emerging evidence has suggested the potential role for viruses in the development of periodontal diseases, with particular attention to the Herpesviridae family [13,40]. In detail, current data suggest that periodontal disease may occur more frequently and progress more rapidly in sites harboring herpesvirus infections compared with non-infected ones.

Recent findings indicate that in gingival tissues, microRNAs (miRNAs) encoded by HHVs have been suggested to influence host gene expression profiles, interfering with immune mechanisms, which may contribute to persistent inflammation and tissue destruction. As post-transcriptional regulators, miRNAs are essential for maintaining tissue homeostasis; however, when their expression becomes dysregulated, miRNAs can contribute to pathological conditions, including periodontitis. Herpesvirus-derived miRNAs may disrupt immune equilibrium within the gingival microenvironment by modulating both innate and adaptive immune responses [55].

Herpesviruses can persist in a latent state within infected epithelial cells and leukocytes, evading the innate and adaptive host’s immune responses. Under conditions of stress, immunosuppression, or oral dysbiosis, these viruses may reactivate, amplifying inflammatory responses and periodontal tissue destruction [13,56,57]. Indeed, immune cells infected by Herpesvirus exhibit a reduced capacity to defend the host against pathogenic bacteria.

Of note, periodontal sites harboring herpesviruses are characterized by elevated levels of multiple periodontopathogenic bacteria, such as *Porphyromonas gingivalis*, *Treponema denticola*, *Tannerella forsythia*, *Prevotella intermedia*, *Prevotella nigrescens*, *Campylobacter rectus*, and *Aggregatibacter actinomycetemcomitans (A. a.)* [57].

Among the Herpesviridae, herpes simplex virus type 1 (HSV-1) is one of the most common viruses associated with oral–facial manifestations, infecting approximately 3.5 billion people worldwide [58]. Primary infection is usually acquired in childhood, after which HSV-1 establishes a latent infection in the trigeminal ganglia. Previous studies have detected HSV-1 more frequently in the subgingival plaque and saliva of periodontal pockets in subjects with periodontal diseases, particularly in active sites [58]. Chen et al. [59] hypothesized that the lytic phase of HSV-1 infection enhances the susceptibility of periodontal tissues to colonization by periodontopathogenic bacteria, while bacterial infection, in turn, may trigger the reactivation of latent HSV-1.

Human cytomegalovirus (CMV) has been associated with periodontitis [60], and its reactivation seems to be associated with worsening periodontitis lesions [57]. CMV is capable of infecting periodontal macrophages, fibroblasts, monocytes, and T-lymphocytes [61], and its reactivation may stimulate monocytes to produce inflammatory cytokines and fibroblasts to synthesize collagen [60]. These cellular functional alterations, together with their interactions with periodontal bacteria, may increase the susceptibility to periodontal attachment loss [60].

Given that the prevalence of HSV-1 and CMV infections exceeds 90% in developing countries, with infection commonly occurring during childhood and particularly affecting immunocompromised individuals, elucidating the association of HSV-1, CMV, and periodontal diseases in pediatric subjects is of critical importance [60,62].

Epstein–Barr virus (EBV), a double-stranded DNA virus, is transmitted through saliva and may establish a typically asymptomatic, lifelong latent infection. EBV type 1 (EBV-1) is frequently detected in the blood sample of systemically healthy subjects, while EBV type 2 (EBV-2) is more commonly found in immunosuppressed subjects—in particular, in HIV-positive and transplant subjects [63]. EBV-2 causes more B-lymphocyte lysis compared to EBV-1 [63]. EBV is capable of infecting periodontal macrophages and B-lymphocytes [61]. It was detected in periodontal diseases, where the interactions between EBV and bacterial pathogens have been hypothesized to contribute to the progression of periodontal disease, although direct causal links remain to be confirmed [13,56,57].

Varicella Zoster Virus (VZV), an alpha-herpesvirus, typically infects children between 5 and 9 years of age, producing self-limited and benign manifestations in systemically healthy pediatric subjects. In contrast, in systemically compromised pediatric subjects, several complications may occur, such as secondary bacterial infections, pneumonia, cerebellar ataxia, encephalitis, or Reye’s syndrome [64].

Although investigated in previous studies, VZV was never retrieved in the periodontal pocket of both systemically healthy and compromised pediatric subjects with periodontal diseases [18,21]. This may be consistent with the VZV preference to infect epithelial cells, resulting in vesicles on the oral mucosa [64].

### 4.3. Periodontal Fungi in Pediatric Subjects

Fungal species, particularly *Candida* spp., are commensal and opportunistic microorganisms that most commonly colonize healthy oral mucosa and, together with bacteria and viruses, form dental plaque [65]. These opportunistic microorganisms can persist in oral niches without causing clinical symptoms. Instead, the overgrowth of *Candida* spp. may be triggered by an oral environmental change or may be caused by the host’s immune dysregulation (e.g., in cases of diabetes mellitus or immunocompromised disorders) [66].

Previous studies have suggested an association between *Candida* spp. and the progression of chronic periodontitis [67]. In particular, *Candida albicans*, followed by *Candida glabrata*, *Candida dubliniensis*, *Candida tropicalis*, *Candida krusei*, and *Candida parapsilosis*, were the most common and abundant fungal species in periodontal pockets, both in systemically healthy and compromised subjects, especially in periodontal pockets with PPD ≥ 5 mm [68,69].

Furthermore, the crevicular fluid and the periodontal pocket are considered favorable environments for the hyphal tip growth of *Candida albicans* and its germination [67]. In periodontal disease, *Candida* spp. may contribute both by invading the periodontal tissues through the production of toxic metabolites and by cross-interacting with periodontal bacterial pathogens [69].

Other non-*Candida* fungi detected in periodontal pockets include *Aspergillus fumigatus*, *Rhodotorula* spp., and *Saccharomyces cerevisiae* [69].

### 4.4. Periodontal Microbial Interactions in Pediatric Subjects

Periodontal microbial interactions both in systemically healthy and compromised pediatric subjects play a pivotal role in maintaining periodontal health, as well as in the development and progression of periodontal diseases. Table 2 outlines the main inter-kingdom and virus co-infection interactions between bacteria, *Herpesviruses*, and fungi, which are implicated in periodontal disease development and progression in pediatric subjects.

As shown in Table 2, the interactions between fungi and the virus kingdom were not investigated.

## 5. Periodontal Profile in Pediatric Subjects with a Healthy Periodontium

A healthy periodontium reflects a state of dynamic equilibrium, where a symbiotic microbial community coexists with a finely tuned host immune response [73].

Clinically, this is represented by PPD < 3 mm, bleeding on probing < 10%, no clinical attachment loss, no alveolar bone resorption, and stable gingival architecture [3,74].

The microbial composition in this state is predominantly characterized by early colonizers, such as *Streptococcus*, *Actinomyces*, *Veillonella*, and *Capnocytophaga* species, with only minimal presence of late colonizers, including *Fusobacterium nucleatum* and *Treponema denticola*, in lower non-pathogenic amounts [38,56]. *Herpesviridae* and *Candida* species may also be present at low, controlled levels, effectively contained by intact epithelial barriers and competent innate immune response [15,75,76].

Host immune factors in periodontal health involve the controlled migration of neutrophils and the basal release of antimicrobial peptides, such as β-defensins and cathelicidins [77,78]. Gingival crevicular fluid (GCF) from healthy sites contains low levels of pro-inflammatory cytokines, including IL-1β and IL-6, which are balanced by anti-inflammatory modulators [79,80,81]. Local factors, including intact epithelium architecture, adequate salivary flow, and effective oral hygiene, support this host–microbe balance [37,82,83], while systemic conditions may perturb this balance [8,84,85].

In pediatric populations, the simultaneous development of the immune system and oral microbiome may confer enhanced resilience [86]. It may be plausible that this co-maturation fosters immune flexibility, facilitating effective microbial containment with minimal tissue impact. GCF markers remain low in health or may increase modestly following transient gingivitis, evidencing early immune engagement without irreversible damage [86,87].

### 5.1. Periodontal Profile in Systemically Healthy Pediatric Subjects with a Healthy Periodontium

In systemically healthy pediatric subjects, the initial colonization is mediated by *Streptococcus* species [88,89]. Interestingly, even in the absence of periodontal disease, anaerobic and proteolytic bacteria commonly associated with dysbiosis, such as *Fusobacterium nucleatum*, *Prevotella intermedia*, *Porphyromonas gingivalis*, and *Treponema denticola*, can be detected, although with lower frequency and relative abundance.

Indeed, in salivary and/or subgingival samples from systemically healthy children with clinically healthy periodontium, several late colonizers were identified, in particular *Fusobacterium nucleatum* (47.37%), *Prevotella intermedia/nigriscens* (36.84%), *Porphyromonas gingivalis* (29.7%), and *Tannerella forsythia* (25.00%) [21]. These findings suggest that the presence of species belonging to late biofilm colonizers alone might not inevitably result in disease, but rather reflects an ecologically controlled coexistence shaped by host immunity and environmental factors.

Moreover, *A. a.* was identified in 14.9% of systemically healthy children [21]. This species is of particular interest due to its heterogeneity: while highly virulent strains, such as the JP2 genotype, are strongly associated with aggressive periodontitis, non-JP2 genotypes have been isolated from healthy sites and are generally considered compatible with periodontal health [90,91]. It might be plausible that the low prevalence observed in pediatric healthy individuals is attributable to colonization by non-pathogenic variants, whose presence does not disrupt homeostasis but may even contribute to microbial diversity. Notably, microbial composition evolves throughout childhood in tandem with dentition stages [92]. In deciduous dentition, taxa such as *Pseudomonaceae*, *Enterobacteriaceae*, and *Aggregatibacter* are detected, whereas the transition to mixed and permanent dentition is marked by increasing abundance of *Veillonella*, *Prevotella*, *Bacteroidetes*, and *Spirochaetes* [92]. Puberty introduces further microbial shifts, driven by hormonal modulation and increased nutrient availability, which favor the colonization of anaerobic Gram-negative species [92]. Moreover, the “microbial priming” during early childhood implies that certain taxa may transiently inhabit the periodontal niche, contributing to the host’s immunological modulation, without necessarily eliciting tissue destruction [93]. It might be plausible that in systemically healthy pediatric individuals, the periodontal niche maintains an immunologically tolerant but vigilant state, in which microbial metabolites, including short-chain fatty acids, proteases, and extracellular vesicles [56,94], are constantly surveyed but contained within a homeostatic network.

Immune homeostasis within the periodontal niche is maintained through the balanced activation of pattern recognition receptors such as Toll-like receptors (TLRs), which recognize microbial-associated molecular patterns and coordinate controlled neutrophil recruitment while preserving epithelial integrity [95]. This response is finely tuned—neither excessive nor passive—to tolerate commensal microorganisms while limiting the expansion of pathobionts.

Moreover, the functional profile in a healthy oral microbiome could be related to the enrichment in genes related to carbohydrate metabolism and anti-inflammatory molecules biosynthesis, in contrast to the proteolytic, virulence-associated gene signatures of dysbiosis [96]. Although the bacterial community remains the most extensively characterized component of the oral microbiome, emerging evidence indicates the presence of a structured oral virome even in systemically healthy children [21,97]. Bacteriophages are believed to contribute to microbial equilibrium by regulating bacterial population dynamics through lytic or lysogenic cycles [97,98], and eukaryotic viruses, especially those belonging to the *Herpesviridae*, *Papillomaviridae*, and *Anelloviridae* families, were found in the oral microbiome of asymptomatic healthy subjects [37].

In particular, although most pediatric subjects with a healthy periodontium tested negative for HHVs, a small proportion showed a viral presence [21].

HSV-I was the most detected virus among this population [21], potentially reflecting asymptomatic primary infection common in early childhood. It might be plausible that detection in a clinically healthy periodontium represents a latent or preclinical phase, preceding the symptomatic reactivation typically observed as labial herpes lesions. Similarly, VZV was not observed in this cohort [21], suggesting a limited affinity for the periodontal niche or an effective immunological clearance by the host. CMV was reported in 17.80% and EBV in 22.09% of children with a healthy periodontium [21]. Although CMV and EBV may exhibit tropism for the sulcular and junctional epithelium [38], their presence in the healthy periodontium may be associated with the latency or non-transcriptional state of Herpesviridae or to the proper immune response of the host [40]. Indeed, CMV is known to evade immune detection by interfering with natural killer cell recognition [99], yet the intact immune function in systemically healthy children, both humoral and cell-mediated, may have sufficed to prevent reactivation and maintain a clinically quiescent state.

Fungal profiling in children with a clinically healthy periodontium is limited. However, existing literature suggests a limited colonization by *Candida* species (*C. albicans*, *C. tropicalis*, and *C. glabrata*) in periodontal health [100]. In a study on adolescents undergoing orthodontic therapy, *Candida albicans* was significantly less prevalent in subjects with a healthy periodontium. In those with gingivitis [101], there is likely a lower biofilm mass, reduced ecological complexity, and limited fungi interactions with bacteria or viruses, and it is likely held in check by salivary defenses, antimicrobial peptides, and mucosal immunity.

These findings support the view that clinical periodontal health in children corresponds to a regulated microbial architecture, comprising bacteria, viruses, and fungi, that is surveilled and shaped by a responsive and calibrated immune system [102,103].

### 5.2. Periodontal Profile in Systemically Compromised Pediatric Subjects with a Healthy Periodontium

The periodontal profile of systemically compromised pediatric subjects with a clinically healthy periodontium is poorly and sparsely characterized in the available literature and limited to select conditions [25,104,105,106,107]. However limited, though, these findings may offer valuable insights regarding the dynamic relationship between microbial colonization and systemic disease in shaping periodontal health.

For instance, Lyko et al. [106] reported detectable levels of *Capnocytophaga* spp., *Prevotella* spp., and *Fusobacterium nucleatum*, while other periodontal pathogens, such as *A. a.* and *Porphyromonas gingivalis*, were either absent or significantly less prevalent compared to those with established periodontal disease.

In children with Down syndrome, Vocale et al. [105] revealed that even in the deciduous dentition, these individuals may harbor a higher prevalence of well-recognized periodontopathogens—including *P. gingivalis*, *T. denticola*, and *A. actinomycetemcomitans*—compared to age-matched, systemically healthy peers. Nonetheless, the absence of periodontal disease in many subjects suggests that the mere detection of pathogens is insufficient to trigger disease onset. It might be plausible that these microbial patterns represent an early or pre-dysbiotic phase, in which colonization by periodontal pathogen bacteria may occur without the concurrent inflammatory or immunological cofactors required to initiate tissue damage. Collectively, these observations support the concept that microbial presence should be interpreted within the broader host–microbe–immune context.

Similarly, despite quantitative differences, microbial profiles of children with type 1 diabetes mellitus and healthy periodontal tissues were largely comparable to those from healthy controls [107]. This may suggest that well-managed glycemic control and close medical monitoring prevent microbial dysbiosis, despite underlying metabolic stress. In HIV-positive children, the absence of periodontal disease reported in some cases [25] suggests a degree of immune restoration that restrains both dysbiosis and viral reactivation. In these individuals, immune dysfunction—marked by reduced CD4+ T-cell counts and persistent immune activation—may justify the reported presence of HHVs, such as CMV and EBV. Moreover, it should be considered that HHVs have the capability to remain latent or exhibit only transient low-level replication [108,109], which may be insufficient to trigger pathological tissue breakdown. Furthermore, in HIV-positive subjects under HAART-driven immune recovery, the HHVs’ activity may be stabilized or present at subclinical levels [110,111], potentially limiting their impact on the periodontal niche.

A similar scenario could be applied to pediatric subjects affected by malnutrition or kwashiorkor, in whom the detected presence of *Herpesviridae* [26] is likely attributable to immunosuppression. In these children, the reported absence of periodontal disease [26] despite systemic immunodeficiency might be explained by insufficient pro-inflammatory bacterial biofilms capable of co-activating viral reactivation and eliciting destructive immune responses [46,112]. It might be hypothesized that without the provision given by the bacterial biofilm, together with the host immune response, herpesviruses remain silent bystanders. For instance, CMV was detected in 17.8% of systemically healthy subjects and 15.09% of those with systemic diseases [18], indicating that its presence may be more dependent on local mucosal factors than on systemic immune status [21].

The predominance of HHV-negative findings in systemically compromised pediatric cohorts [18]—especially for HSV, CMV, and EBV-I—supports the idea that co-pathogenic microbial conditions might be required to unlock the pathogenic potential of these viruses. Moreover, the absence of viral co-infections in children with a healthy periodontium [18] suggests that the microbial environment plays an essential role not only in supporting viral persistence but also in determining their immunological impact. In the absence of a dysbiotic biofilm enriched with periodontal pathogenic bacteria, HHVs alone are unlikely to initiate periodontal disease. Rather, they appear to function as latent cofactors, reactivating primarily in the context of bacterial-driven inflammation or profound immune dysregulation [18]. It might be plausible that certain herpesviruses attenuate excessive immune responses or confer cross-protection against bacterial colonization, ultimately contributing to periodontal homeostasis [14,113]. In children with systemic conditions, the relative negativity for both bacterial and viral pathogens may also result from pharmacological modulation, altered mucosal immunity, or ecological shifts that reduce the availability of microbial cofactors required for synergistic pathogenesis [114,115].

Similarly, although less investigated, fungi were absent or less represented [107] in these subjects, which suggests either a lack of fungal overgrowth or effective ecological suppression [72,103].

**Figure 2:**
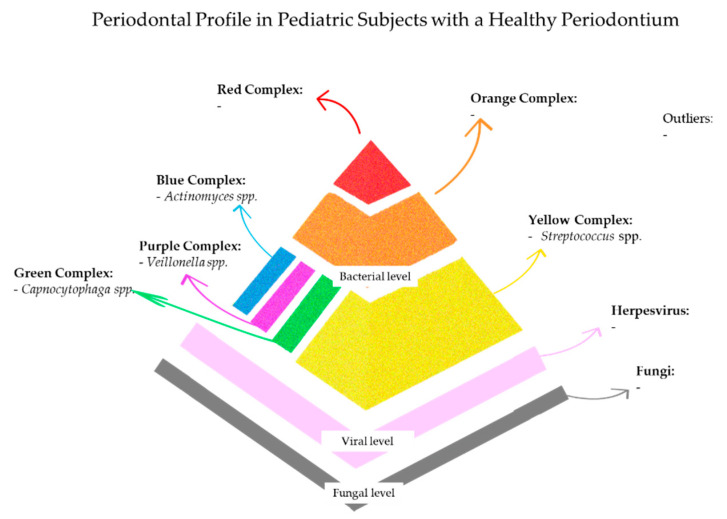
Periodontal profile in healthy and systemically compromised pediatric subjects with a healthy periodontium: associated bacterial (according to Socransky’s complexes), viral, and fungal composition.

Figure 2 summarizes the main microbial composition of the periodontal profile in pediatric subjects with a healthy periodontium.

## 6. Periodontal Profile in Pediatric Subjects with Gingivitis

Gingivitis is reversible and the most common periodontal disease among children [116], characterized by the absence of CAL and radiographic bone loss, and by the presence of bleeding on probing (BoP) at almost 10% of sites [117]. If gingivitis persists, it may lead to periodontitis, particularly in susceptible individuals [118]. Chronic gingivitis is more prevalent among 12–19-year-old adolescents, especially in orthodontic subjects [119], in which a relatively poor plaque accumulation may trigger a gingival inflammation reaction [120,121]. However, the risk of progression in periodontitis is lower in young adolescents than in adults [122].

Although numerous epidemiological studies have demonstrated that gingivitis is a common periodontal disease, considerable variability has been reported in the recorded prevalence rates, which can be explained by considering socio-demographic factors, oral hygiene behaviors, inter-population variability, and the children’s systemic health status—healthy or systemically compromised, which increases the susceptibility to gingivitis—and the heterogeneity in criteria used to assessed diagnosis (e.g., based on the community periodontal index, gingival index, or the latest 2017 World Workshop criteria) [123].

The reported prevalence of gingivitis in pediatric subjects (<18 years old) varied from 91% in Romania [124] to 77% in Brazil [125] and 29.65% in Southern China [123].

### 6.1. Periodontal Profile in Systemically Healthy Pediatric Subjects with Gingivitis

In systemically healthy pediatric subjects with gingivitis, the bacterial profile is often characterized by species associated with periodontal disease [126].

Early biofilm formation is dominated by *Streptococcus sanguinis* and *Actinomyces* spp., which establish a foundational environment facilitating the subsequent colonization of anaerobic species implicated in gingival inflammation [126]. Among these, *Prevotella intermedia*, *Fusobacterium nucleatum*, *Campylobacter rectus*, *Peptostreptococcus micros*, and *Capnocytophaga* spp. were found in gingivitis, and their abundance was related to clinical signs of inflammation, such as BoP and elevated gingival index scores [127].

Moreover, higher values of the Plaque Index (PI) created a conducive environment for the proliferation of anaerobic and proteolytic species, which provides the substrate for pathogenic bacteria and exacerbates local inflammation [127]. Consequently, PI is positively linked with both microbial diversity and inflammatory indices [127,128], suggesting that mechanical plaque control in children is crucial for modulating microbial ecology and interrupting the progression toward gingival inflammation and potential progression to periodontitis.

In gingivitis, pediatric subjects with increased levels of *Prevotella intermedia* and *Fusobacterium nucleatum* showed pronounced marginal erythema and bleeding [128], supporting the hypothesis that specific “keystone” pathogens can drive early local inflammatory amplification before establishing deeper periodontal disease. Additionally, these bacteria promote the production of pro-inflammatory cytokines, which in turn enhance vascular permeability and recruit neutrophils, clinically manifesting as increased BoP and sulcus bleeding index [128,129].

Although these pediatric subjects have yet to exhibit CAL or periodontal pockets, the concurrent presence of increased PI, specific anaerobic bacteria, and the release of proteolytic enzymes and virulence factors capable of tissue damage may predispose them to potential periodontal disease progression. Moreover, salivary biomarkers such as MRP-8/14, elevated in these cases, further suggest an ongoing inflammatory response that could predispose to connective tissue degradation and alveolar bone resorption if not adequately controlled [130].

In pediatric subjects with gingivitis, among *Herpesviridae*, HSV-1 was reported in 18.75% of cases [21]. HSV-1 has an affinity for oral and sulcular epithelial cells, the ability to disrupt intercellular junctions, and the capacity to promote vascular infiltration by inflammatory cells [40,58]. Its confined replication to the superficial epithelium may also explain the reported association with gingivitis rather than periodontitis, where connective tissue and deeper inflammatory pathways are involved [21].

Conversely, CMV was not associated with gingivitis cases [21], potentially reflecting its affinity to more severe forms of periodontal disease. Indeed, CMV interacts with Toll-like receptors (TLRs), including TLRs 2, 7, and 9, which are upregulated in periodontitis lesions compared to gingivitis [21,40].

Moreover, the reported absence of VZV in children affected by gingivitis [21] might further reflect its tropism for cutaneous epithelium rather than oral mucosa.

Furthermore, the children’s systemic health status may have impacted their ability to control viral infections, leading to a greater proportion of HHV-negative cases [21].

The role of fungi in pediatric gingivitis is underreported and debated [21]; however, growing evidence [131] suggests their potential involvement in early inflammation.

A recent study [131] analyzed fungi in supragingival sites of gingivitis in adolescents undergoing orthodontic treatment. Among them, *Candida albicans* and *Candida dubliniensis* were the most frequently encountered fungi, with a lower abundance of *Saccharomyces cerevisiae*, *Malassezia restricta*, *Cladosporium* spp., and *Aspergillus* spp. [131]. Fungi, particularly *Candida* species, contribute to immune activation through epithelial disruption, hyphal penetration, and the release of virulence factors such as secreted aspartyl proteinases and *Candida* lysin [131].

### 6.2. Periodontal Profile in Systemically Compromised Pediatric Subjects with Gingivitis

The microbiological profile in systemically compromised pediatric subjects with gingivitis is generally poorly characterized and only reported in certain conditions (Down syndrome, type 1 diabetes mellitus, juvenile idiopathic arthritis, and HIV infection) [10,16,18,25,66,82].

Bacterial communities in systemically compromised children commonly exhibit an increased amount of key periodontal pathogens, including *Fusobacterium nucleatum*, *Campylobacter rectus*, *Porphyromonas gingivalis*, *Treponema denticola*, and *Prevotella* spp. (*P. intermedia* and *P. nigrescens*) [10,82]. For instance, in children with Down syndrome, BoP was associated with a higher abundance of these pathogens, with an intriguing age-related distribution: *F. nucleatum*, *P. intermedia*, and *P. nigrescens* were more abundant in the 3–7-year age group, while *C. rectus* peaked in younger children aged 8–12 years [82].

Similarly, in children with type 1 diabetes mellitus, persistent hyperglycemia may increase inflammatory cytokine production and impair leukocyte function, creating a favorable environment for microbial dysbiosis. Notably, among these patients, *Capnocytophaga sputigena* and *Capnocytophaga ochracea* from the green complex were more prevalent in gingival sites, and *F. nucleatum* and *P. nigrescens* showed strong co-occurrence, indicating their potential role in early periodontal pathology [10]. Additionally, poor metabolic control was correlated with higher bacterial loads, further reinforcing the bidirectional relationship between systemic disease and oral microbial communities [10].

In children affected by juvenile idiopathic arthritis, the presence of periodontopathogenic species and BoP scores was reported to be unaffected by medication or disease activity [16]. This finding may potentially suggest that alterations in the oral microbiota may play a direct role in driving both local gingival inflammation and systemic immune dysregulation. Moreover, immunosuppressive therapy may likely allow the expansion of pathogenic microbial communities that are normally kept in check by the host’s proper immune defenses.

In systemically compromised pediatric patients with gingivitis, the viral profile revealed a higher prevalence of HSV-1 compared with other periodontal conditions [18], potentially suggesting that HSV infections may preferentially occur in periodontal conditions affecting primarily the superficial epithelial layers, which may be conducive to viral replication, as opposed to conditions like periodontitis that involve deeper tissue structures.

Moreover, the detection of CMV among HIV-positive children with gingivitis [18,25] further indicates that chronic immunosuppression in these patients likely facilitates viral reactivation and diminishes the capacity to control infection or clear latent viruses. Indeed, in HIV-infected children, viral replication and immune suppression may synergistically enhance susceptibility to secondary infections, including both bacterial and fungal pathogens. It may be plausible that chronic viral infections perpetuate a cycle of inflammation and immune dysregulation, thereby accelerating gingivitis progression and potentially its evolution into periodontitis.

**Figure 3:**
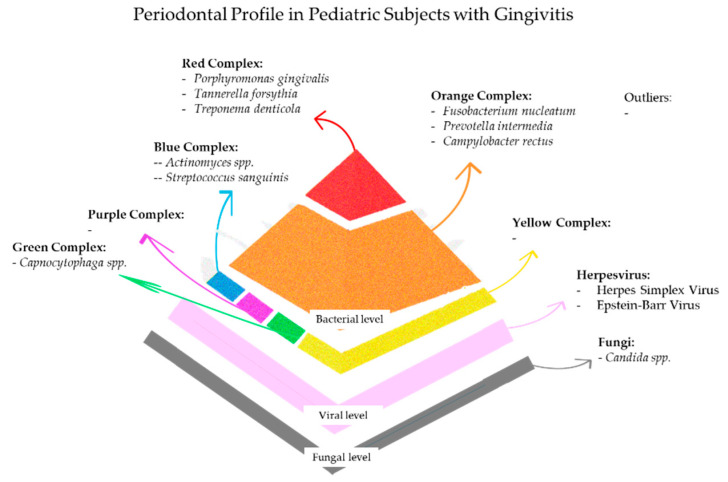
The periodontal profile in healthy and systemically compromised pediatric subjects with gingivitis and the associated bacterial (according to Socransky’s complexes), viral, and fungal composition.

The role of fungi in the pathogenesis of gingivitis remains largely unexplored, particularly in pediatric populations with systemic comorbidities. While no direct data on *Candida* spp. in gingivitis are currently available, several studies have documented a significantly higher oral carriage of *Candida albicans* and other non-*albicans* species in systemically compromised children, especially in children with Down syndrome, compared to healthy controls [66]. In these individuals, *C. albicans* colonization is frequently associated with lip fissures and erythematous candidiasis, particularly in younger age groups, and may be facilitated by salivary alterations, immune dysfunction, and coexisting mucosal abnormalities [66]. These factors may likely create a permissive environment for fungal persistence and overgrowth even in the absence of overt mucosal lesions. Moreover, the high prevalence of enzymatically active fungal strains in children with Down syndrome may indicate that Candida may express increased virulence potential in this context, even in clinically non-candidal presentations; however, its contribution to gingival inflammation remains unclear. Figure 3 summarizes the main microbial composition of the periodontal profile in pediatric subjects with gingivitis.

## 7. Periodontal Profile in Pediatric Subjects with Necrotizing Gingivitis/Periodontitis

Necrotizing gingivitis and periodontitis are rapidly progressing inflammatory periodontal diseases characterized by interdental papillae necrosis, pain, and spontaneous bleeding [3,6,7]. It is typically observed in individuals with poor oral hygiene, immunocompromised status (especially in cases of HIV infections), or malnutrition [3,6,7]. In such patients, or when necrotizing gingivitis is not promptly treated, the disease may rapidly progress to necrotizing ulcerative periodontitis [132].

Young adults are more commonly affected than children (aged 2–7 years), who, however, when affected, generally exhibit more severe manifestations [132].

Necrotizing gingivitis and periodontitis are associated with fusiform and spirochetal bacteria and represent a rare form of periodontal disease in which the use of systemic antibiotics is recommended for managing the acute phase. These conditions constitute the third most frequent periodontal disease for which dentists prescribe antibiotics [133], occasionally in combination with antifungal therapy in immunocompromised patients.

### 7.1. Periodontal Profile in Systemically Healthy Pediatric Subjects with Necrotizing Gingivitis/Periodontitis

Notably, no previous studies had comprehensively investigated and reported the bacterial, viral, and fungal periodontal microbial profiles in systemically healthy pediatric subjects affected by necrotizing gingivitis/periodontitis. Meanwhile, such conditions were absent in the systemically healthy subjects [21]. This lack in the literature suggests that systemic diseases increase the risk of developing necrotizing gingivitis/periodontitis, leading to more severe periodontal diseases due to impaired host immune systems and a subsequent amplified inflammatory response, which exacerbates periodontal tissue damage [134].

### 7.2. Periodontal Profile in Systemically Compromised Pediatric Subjects with Necrotizing Gingivitis/Periodontitis

The bacterial periodontal profile of necrotizing gingivitis/periodontitis is specifically characterized by spirochetes like *Treponema* spp. and *Fusobacteria*, *Prevotella intermedia*, *Selenomonas* spp., which were called the “constant flora,” as well as *Porphyromonas gingivalis* and *Peptostreptococcus* spp. [6,135]. However, this knowledge is primarily derived from previous studies in adults, while research on the bacterial profile in pediatric subjects is limited.

The viral periodontal profile characterized in systemically compromised pediatric subjects affected by necrotizing gingivitis showed a higher prevalence of HSV (16.67% of tested samples) compared to systemically compromised pediatric subjects affected by periodontitis [18], probably because HSV preferentially affects the superficial epithelial layers [38].

In systemically compromised pediatric patients, necrotizing gingivitis represented the periodontal condition with the highest detection rate of EBV-1 (20.69% of tested samples), followed by periodontitis [18]. However, the lower detection rates of EBV in samples from systemically compromised pediatric subjects with necrotizing gingivitis compared to those with periodontitis may reflect a preferential EBV tropism for deeper periodontal tissues. This is consistent with EBV’s known ability to persist in infected B-lymphocytes at the sulcular and junctional epithelium [136,137].

In Down syndrome, the increased activity of matrix metalloproteinase and compromised T-cell function increases the inflammatory reaction and tissue destruction [8,137,138], facilitating EBV reactivation; in Papillon–Lefèvre and Kostmann syndromes, functional neutrophil deficiencies and compromised antimicrobial defenses may favor chronic inflammation and viral reactivation [8]; and in Hydroa vacciniforme, an EBV-associated systemic disease characterized by a lymphoproliferative disorder of T-cells [139], the macrophages that EBV infects in periodontal tissues may maintain a chronic inflammation and lead to consequent periodontal tissue destruction.

Also, CMV was detected in pediatric subjects affected by malnutrition/kwashiorkor (19.40% of tested samples) [18], both diminishing the essential nutrients and altering the host immune system’s function and the related response against HHV infections [108]. Interestingly, in systemically compromised pediatric subjects with periodontal acute conditions such as necrotizing gingivitis, both HSV and CMV were more prevalent.

Finally, HHV-6 was a rare detection in necrotizing gingivitis of systemically compromised pediatric subjects (4.35% of tested samples), while HPV was not retrieved in any samples [18]. This finding was unexpected given the high prevalence of latent HPV infection in the general population, affecting both healthy and systemically compromised individuals. In contrast, productive HPV infections—manifesting in the oral cavity more frequently as benign lesions such as papillomas, verruca vulgaris, condyloma acuminatum, and focal epithelial hyperplasia—show higher prevalence rates in low-income countries such as Nigeria, where malnutrition and kwashiorkor are common among pediatric populations [140].

Coinfections involving two or three types of HHVs were detected in 9.09% and 27.27% of the analyzed samples, respectively. The detection of different types of HHVs in gingival tissues indicates that the gingiva may be a latent reservoir for HHVs [58,141,142]. Furthermore, given the inability of an adequate host immune response, increased virulence causes more severe clinical manifestations, resulting in acute periodontal diseases like necrotizing gingivitis.

The fungal periodontal profile has not been extensively investigated in previous studies. However, it is noteworthy that subjects affected by necrotizing gingivitis are often systemically compromised, suggesting that the presence of *Candida* spp. may be conditioned by the host’s immunosuppression rather than a primary etiological factor. Nevertheless, the microbial profile in necrotizing gingivitis/periodontitis in HIV-positive subjects, characterized by bacterial and *Candida albicans* superinfections, as well as co-infection with HHVs, appears to resemble the microbial profile of HIV-negative subjects with periodontitis [135].

## 8. Periodontal Profile in Pediatric Subjects with Periodontitis

Periodontitis is a multifactorial, chronic, plaque-associated, inflammatory disease characterized by a progressive and irreversible destruction of periodontal tissues, identified by a clinical attachment loss higher than 2–3 mm at almost two non-adjacent teeth [3].

Periodontitis in pediatric subjects is relatively uncommon compared to in adult subjects [116]. The prevalence of periodontitis among children and adolescents varies from 2 to 6%, depending on demographic, genetic, geographic, and socioeconomic variables [143]. In fact, “chronic periodontitis” is the subdivision of periodontitis, generally characterized by a slow destruction of the periodontal tissues, and is most common in adult subjects [3]. In contrast, another subdivision of periodontitis is “aggressive periodontitis,” a rapid, destructive form, usually affecting pediatric subjects, prior classified as “rapidly progressive periodontitis” or “early-onset periodontitis” [3]. Aggressive periodontitis typically has a clinically well-recognized extension and distribution limited to a molar–incisor pattern, which suggests a distinct pathophysiology (microbiology, genetic background, host immune response) that is still poorly understood [3]. Finally, periodontitis in children is frequently associated with systemic factors (e.g., gender, hormones, ethnicity), systemic diseases (e.g., Down syndrome, malnutrition, immunological disorder), or local conditions (e.g., plaque accumulation, dental anomalies, orthodontic appliances) [143,144]. A subdivision of periodontitis comprises “periodontitis as a manifestation of systemic disease,” which comprises several pathological systemic conditions in which periodontitis is a manifestation of the disease [3].

### 8.1. Periodontal Profile in Systemically Healthy Pediatric Subjects with Periodontitis

Bacteria of Socransky’s red complex, including *Porphyromonas gingivalis*, *Tannerella forsythia*, and *Treponema denticola*, and of Socransky’s orange complex, such as *Prevotella intermedia*, *Fusobacterium nucleatum*, and *Campylobacter rectus*, are associated with periodontitis diseases [41]. Recent research has further highlighted that certain bacteria, such as Tannerella forsythia, are more prevalent in periodontal pockets affected by periodontal diseases, and this correlation is associated with specific periodontal parameters [118,145].

Although pathogenic bacteria were also detected in the healthy periodontium of systemically healthy pediatric patients, cases of periodontitis demonstrated a broader spectrum of species and a higher prevalence. In particular, in those with generalized forms, the highest prevalence of bacterial species found was *Treponema denticola* (70.59%), followed by *Porphyromonas gingivalis* (55.40%), *A. a* (36.58%), and *Tannerella forsythia* (35.90%) [21]. Unexpectedly, with the exception of *Treponema denticola*—which exhibited a higher prevalence in systemically healthy pediatric patients with a healthy periodontium (82.35%)—the remaining bacterial species showed higher prevalence in diseased sites. Additionally, in systematically healthy pediatric subjects affected by periodontitis and positive for *A. a.*, higher bone loss was recorded compared to those who were negative [112]. These findings suggest that *Porphyromonas gingivalis* and *A. a.* cannot alone explain periodontal disease.

The bacteria most frequently detected in pediatric subjects affected by molar–incisor pattern periodontitis (MIPP) included *Fusobacterium nucleatum* (95.95%), *Campylobacter rectus* (72.73%), *Treponema denticola* (70.59%), *A. a.* (19.51%), and *Porphyromonas gingivalis* (18.92%). The specific role of *A. a.* in the pathogenesis of MIPP, especially the Jp2 genotype serotype b, is well established in young subjects [112,146]. In particular, the presence of Jp2 genotype *A. a.* was associated with aggressive progression and worsening of clinical parameters, such as CAL or PPD [90]. Nonetheless, in periodontal pockets with higher PPD, a higher total bacterial count was registered [90].

In systemically healthy pediatric subjects with periodontitis, the viral profile of HHVs has been detected at varying prevalence rates depending on the clinical form of the disease [21], suggesting a potentially distinct virological burden in generalized periodontitis compared to MIPP.

In generalized periodontitis cases, CMV (36.36%) and EBV-1 (36.24%) were the most identified herpesviruses, with co-infections involving multiple HHVs also being more common, including CMV and HSV (12.5%) and CMV and EBV-1 (24.24%) [21]. A smaller proportion of generalized periodontitis also harbored EBV-2 (6.25%) [21]. By contrast, MIPP showed a considerably lower viral prevalence [21]. Nonetheless, also in MIPP, CMV and EBV-1 remained the most frequently detected viruses, identified in 13.64% and 10.14%, respectively, while coinfections were rare or absent [21]. These viruses are capable of persisting in a latent state within host cells, including gingival fibroblasts [147]. Under specific inflammatory or environmental stimuli, such as sustained immune activation, hormonal fluctuations, or psychosocial stress, latent *Herpesviridae* can shift into a replicative phase [13]. Viral reactivation is often accompanied by the production of pro-inflammatory mediators, including interleukin-1β and tumor necrosis factor, which amplify the local inflammatory response and promote osteoclast differentiation [13], potentially implicating periodontal tissue destruction, especially when the immune system is unable to regulate the inflammatory response effectively.

It is conceivable that the higher frequency of HHVs detected in generalized periodontitis, compared to MIPP, might be explained by a broader and more persistent inflammatory microenvironment and a larger number of affected sites.

Moreover, the virological profile found in systemically healthy children with periodontitis aligned with the severity of clinical periodontal parameters found [21]. Indeed, PPD ranged from 5 to 9 mm, with a mean of 6.9 ± 1.5 mm in generalized periodontitis and 7.2 ± 0.9 mm in MIPP. Likewise, CAL reached values up to 4.8 ± 1.2 mm in cases testing positive for HHVs [21]. Notably, certain HHVs, including EBV, have been associated with periodontal disease, especially in sites with increased pocket depths and greater clinical attachment loss, further supporting their potential role in the pathogenesis of the disease [148,149].

Although the detection of fungi has not been associated with periodontal disease severity in pediatric subjects, Candida albicans and Candida dubliniensis were found in higher levels compared to healthy sites in Moroccan children [150]. Considering that candidal hyphae are most commonly found in subgingival specimens from systemically compromised subjects [103], the absence of fungi in systemically healthy subjects was likely to be found. Nonetheless, fungi transition from commensal to pathogen may be observed under dysbiotic conditions, as occurs in periodontitis [72,103,150].

### 8.2. Periodontal Profile in Systemically Compromised Pediatric Subjects with Periodontitis

Bacterial diversity was reduced compared with that observed in systemically healthy pediatric subjects, which could suggest a compromised ability to maintain the balanced oral eubiosis due to the host’s immune system dysregulation [21]. This may be manifested in more severe periodontitis, supported by a subgingival biofilm characterized by a lower bacterial diversity but potentially higher bacterial virulence [18,85,151].

A distinct bacterial profile emerged in systemically compromised pediatric subjects influenced by their immunological or metabolic alterations associated with systemic disorders. A consistent presence of bacteria associated with periodontitis of Socransky’s red and orange complexes (in particular, Prevotella intermedia/nigriscens, Fusobacterium nucleatum) was observed across multiple systemically compromised groups [18,39,152,153,154].

In pediatric subjects with periodontitis and DOCK8 deficiency, in addition to these microorganisms, other bacterial species, such as *Capnocytophaga* spp. (*C. gingivalis*, *C. orchea*, *C. sputigena*), were detected [155].

In contrast, *Porphyromonas gingivalis* was not detected in pediatric patients with Kostmann syndrome, a disorder characterized by severe neutropenia [156], who frequently tested positive for *Prevotella intermedia*, also commonly found in Papillon–Lefèvre syndrome. The ability of *Prevotella intermedia* and *Prevotella nigriscens* to form biofilms and tolerate oxidative stress may have a particular advantage, especially in environments with diminished neutrophil activity [151,156,157].

Meanwhile, *A. a.* was present in subjects affected by Fanconi anemia and diabetes mellitus [39,90,112]. Interestingly, *A. a.* was absent in children affected by Kostmann syndrome, suggesting that this bacterium uses metabolic imbalances to its advantage. Thus, neutropenia alone may not constitute a suitable ecological niche for *A. a.* colonization.

In periodontitis, both EBV and CMV were most commonly detected (7.69% and 5.07% of tested samples, respectively), and the highest prevalence was registered for EBV-I. These viruses may play a crucial role in periodontitis, amplifying the inflammatory response and immune activation, as well as the periodontal tissue destruction, interacting with Toll-like receptors largely expressed in periodontally affected sites [18].

In particular, a sample’s positivity to CMV should be investigated if the positive result is for the latent or active form of CMV, which may have a direct role in inflammatory cytokines (like interleukin-1β and TNF-α) and periodontal disease progression, promoting osteoclastogenesis [13]. Nonetheless, the detection of CMV was more common than HSV in severe periodontitis of systemically compromised pediatric subjects.

Other viruses found in children affected by Hydroa Vacciniforme and periodontitis were HHV-6 and HHV-7, which infected CD4+ T lymphocytes, macrophages, monocytes, and fibroblasts [13,58,141,142].

The presence of HHVs in periodontal pockets of systemically compromised pediatric subjects was correlated with worst periodontal clinical parameters [158,159,160]. Through the production of miRNAs by HHVs and the consequent induction of a chronic pro-inflammatory status in pediatric subjects with gingivitis and periodontitis, higher levels of full-mouth bleeding scores were likely recorded. Also, radiographic parameters showed severe bone loss, probably induced by the RANKL/OPG dysregulation induced by HHVs such as CMV and EBV, with an average of more than ⅓ of bone loss in more than 1:2 periodontitis sites [60,136,141,147].

Candidal hyphae were most commonly detected in subgingival samples of systemically compromised subjectsc ompared to systemically healthy ones [103]. However, also in this pediatric population, fungal culture showed that *Candida albicans* represented only 0.3% of the total microbial culture. Moreover, in periodontitis sites, the inflammatory status may facilitate the *Candida albicans* shift to an opportunistic form. However, during the active phase in which an acute inflammatory response occurs in periodontal tissues, fungal growth may be suppressed by the host’s reaction [103].

Among systemically compromised pediatric subjects with periodontitis, *Candida albicans* was detected in children affected by Fanconi anemia and diabetes mellitus. Nonetheless, it was well documented that patients affected by genetic diseases like Fanconi anemia, characterized by host immune disorders and impaired hemopoiesis, are more susceptible to opportunistic fungal infections [8,161,162]. Similarly, diabetic mellitus patients also have a higher risk compared to systemically healthy subjects due to hyperglycemic status, which enhances biofilm accumulation and *Candida*–epithelial cell adherence [103,163]. Additionally, in diabetic patients, fungal colonization may be aided by advanced glycation and products that interact with the RAGE-related receptor and release inflammatory cytokines, generating an oxidative stress that impairs healing and promotes fungal proliferation [8,164,165].

In contrast, fungi were not detected in subgingival sampling of tested pediatric subjects with periodontitis and affected by DOCK8 deficiency or Papillon–Lefèvre syndrome [155,166].

**Figure 4:**
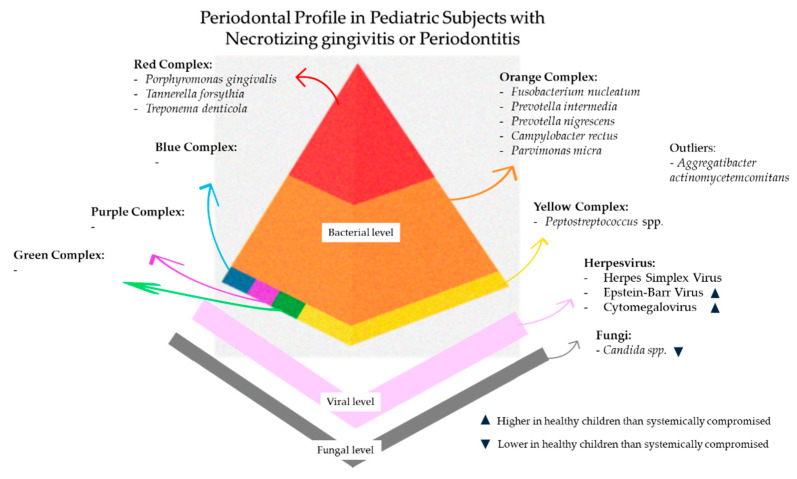
The periodontal profile in healthy and systemically compromised pediatric subjects with necrotizing gingivitis or periodontitis: associated bacterial (according to Socransky’s complexes), viral, and fungal composition.

Figure 4 summarizes the main microbial composition of the periodontal profile in pediatric subjects with necrotizing gingivitis or periodontitis.

## 9. General Health, Periodontal Status, and Related Microbial Profile in Pediatric Subjects

In children with systemic diseases, significant alterations have been observed in the composition and complexity of salivary and subgingival microbial communities [18], with marked distinctions between those with gingivitis, necrotizing gingivitis, or periodontitis and their counterparts with a clinically healthy periodontium.

Indeed, individuals with gingivitis, necrotizing gingivitis, or periodontitis displayed a more diverse and enriched microbial ecosystem, encompassing a broader spectrum of bacterial taxa and a more prominent viral and, occasionally, fungal presence. Conversely, those with periodontal health tended to harbor a less diverse microbial community, albeit less taxonomically rich, which may reflect a microbial equilibrium status, consistent with microbiome homeostasis. Such patterns suggest systemic health may profoundly influence microbial colonization and its regulation. An altered host–microbe interface may facilitate the persistence of opportunistic pathogens, shifting the balance between commensal and pathogenic species and ultimately promoting a dysbiotic state conducive to periodontal breakdown [114,151].

However, while diverse in the taxonomic setup, the microbial profiles related to specific systemic diseases often converged on common pathogenic features, including immune evasion, inflammatory amplification, and tissue degradation. The spectrum of systemic conditions investigated among children encompassed rare primary immunodeficiencies and genetic immune dysregulation syndromes [167,168,169], secondary immune suppression resulting from infectious or nutritional etiologies [104], multisystem genetic syndromes [24], and metabolic and hematologic disorders affecting the immune system [170,171,172,173], outlined in detail below.

### 9.1. Rare Primary Immunodeficiencies and Genetic Immune Dysregulation Syndromes

Among the primary immunodeficiencies investigated in children, DOCK8 deficiency exemplified the profound impact that severe immune dysregulation may exert on host–microorganism interactions [167]. This autosomal recessive condition, characterized by defective cytoskeletal regulation, impaired immune cell migration, and compromised cytotoxic function, may have fostered an environment that markedly attenuated the host’s capacity to contain and clear periodontal microbial colonizers effectively, thus potentially favoring the persistence of opportunistic microorganisms and the establishment of a dysbiotic milieu within the oral microbiome [39,174].

The microbial profile described in DOCK8-deficient individuals with periodontitis included several high-virulence periodontal pathogens, such as *Porphyromonas gingivalis*, *Treponema denticola*, and *Tannerella forsythia* [39,152,153,154,175]. It may be conceivable that the compromised immunological surveillance inherent to DOCK8 deficiency amplified the persistence and pathogenicity of such bacteria, thereby accelerating the periodontal tissue destruction.

In addition to these pathogens, the presence of Capnocytophaga species (*C. gingivalis*, *C. sputigena*, *C. ochracea*) was noted [167]. Although commonly detected within the oral microbiota of individuals with a healthy periodontium [21], including pediatric subjects, their overabundance in DOCK8-deficient individuals may reflect the impaired neutrophil-mediated microbial surveillance, a key immunological feature of the condition. Moreover, the detection of *Campylobacter concisus* in salivary and subgingival samples from pediatric subjects with DOCK8 deficiency [167], a species increasingly associated with mucosal inflammation in both the oral cavity and the gastrointestinal tract [176,177], may provide compelling evidence that dysbiosis in this condition may extend beyond the periodontal niche. Indeed, *C. concisus* strains can colonize the intestinal mucosa, invade epithelial barriers, disrupt tight junctions, and stimulate pro-inflammatory cytokines. In DOCK8 deficiency, where epithelial integrity and immunological containment are compromised, this pathogen may traverse between mucosal compartments, reinforcing the concept of a bidirectional oral–gut axis of dysbiosis and inflammation.

The viral profile reported was also notable [167], as the detection of HSV-1 may reflect impaired T-cell memory and cytotoxic function. Conversely, the absence of EBV and CMV does not necessarily exclude their presence, given the known association of DOCK8 deficiency with EBV-driven lymphoproliferative disorders and CMV-related opportunistic infections [178,179]. This finding suggests the possibility of subclinical or episodic colonization, which may evade detection due to sampling limitations or transient viral load levels.

Although no fungal species were reported, DOCK8 deficiency might be intrinsically associated with TH17 and IL-17 pathway impairments and recurrent mucocutaneous candidiasis [178,179]. Such fungal colonization, even if intermittent, could further compound the microbial burden and inflammatory milieu within the periodontium.

Interestingly, in DOCK8 deficiency, the atopic profile, reflected by high IgE levels and eosinophilia, may further represent a mechanistic bridge linking systemic and periodontal disease. Previous studies have highlighted associations between IgE-mediated conditions, finding increased susceptibility to periodontitis, higher PPD, and more inflammation, compared to healthy controls [180,181]. Indeed, eosinophilic inflammation has the potential to weaken epithelial barriers and alter local immune environments, possibly facilitating the entry and persistence of microbial agents [178,179]. At the same time, the dominance of type 2 cytokines may impair cytotoxic responses, creating a permissive setting for viral latency and reactivation, particularly of *Herpesviridae*. In the context of DOCK8 deficiency, the elevated IgE levels could thus represent a further element reinforcing the systemic condition and periodontal disease link.

Lastly, the involvement of DOCK8 deficiency in cytoskeletal signaling may impair extracellular matrix dynamics, compromising collagen turnover and weakening periodontal connective tissues [178,179]. This fragility, coupled with increased expression of host-derived metalloproteinases and bacterial collagenolytic enzymes such as gingipains [152,153,154], may promote a cascade of matrix degradation and microbial dysbiosis.

It might be plausible that such tissue vulnerability not only exacerbates periodontal damage but also perpetuates a vicious cycle, in which systemic immune dysfunction facilitates local microbial overgrowth, while periodontal inflammation, in turn, contributes to systemic immune activation or barrier disruption. This interplay suggests a potential bidirectional link between systemic disease and periodontal disease.

In rare immunologically compromised conditions, such as Hydroa vacciniforme, an EBV-associated lymphoproliferative disorder, periodontitis was reported in a pediatric subject, in association with a distinct viral profile comprising EBV, HHV-6, and HHV-7 [167]. EBV is known to promote chronic inflammation and persistent immune activation [136,182], while HHV-6 and HHV-7, with their tropism for CD4⁺ and CD8⁺ T cells, may act synergistically in promoting local immune dysfunction, reducing host control over microbial populations, and possibly accelerating the progression of periodontitis. Although bacterial and fungal communities were not assessed in this case [167], it is plausible that such viral-driven immune alterations fostered a mucosal environment permissive to opportunistic colonization, including anaerobic bacteria and fungi. Indeed, herpesvirus-associated immune suppression has been shown to facilitate the outgrowth of periodontal pathogens such as *Fusobacterium nucleatum* and *Prevotella intermedia*, which thrive in inflamed, oxygen-deprived niches and whose abundance correlates with periodontitis severity [183].

In Papillon–Lefèvre syndrome, the reported microbial profile in pediatric subjects with periodontitis was characterized by the presence of *A. a.*, *Fusobacterium nucleatum* and *Prevotella intermedia/nigriscens*, while *Porphyromonas gingivalis* was absent [167]. This distinctive bacterial community may reflect the altered immunological environment shaped by the syndrome’s genetic defect. Mutations in the cathepsin C gene impair the activation of neutrophil serine proteases, which are essential for effective neutrophil functions (chemotaxis, phagocytosis, and resolution of inflammation), thereby severely undermining antimicrobial defenses within the periodontal niche [184]. Thus, this immune defect may not only facilitate microbial dysbiosis but also perpetuate a pro-inflammatory environment that may drive chronic tissue destruction.

Concurrently, the reported presence of CMV and EBV [167] could have further exacerbated immune dysregulation by impairing antigen-specific immune responses and promoting inflammatory cascades [13,136]. Notably, fungal colonization was absent in affected periodontal sites in children, suggesting that the immune environment shaped by cathepsin C gene deficiency may selectively favor bacterial–viral synergy over fungal persistence.

### 9.2. Secondary Immunosuppression Resulting from Infectious or Nutritional Etiologies

In HIV-positive pediatric individuals, chronic CD4⁺ T cell reduction, neutrophil dysfunction, and dysregulated cytokine signaling may profoundly alter mucosal immunity, potentially fostering the proliferation of bacterial species that thrive in inflammatory conditions.

Although bacterial profiling was not thoroughly assessed in HIV-positive children [25], the established association between immunosuppression and periodontal pathogen bacteria colonization by *Porphyromonas gingivalis*, *Tannerella forsythia*, and *Fusobacterium nucleatum* may suggest that these microorganisms can find favorable conditions under HIV-related immune dysregulation [16,172,185].

Members of the *Herpesviridae* family were also identified among HIV-positive children with gingivitis, specifically CMV, HSV-I, and II, whereas EBV and VZV were absent [25]. Remarkably, CMV has also been identified in HIV-positive individuals with a clinically healthy periodontium [25], suggesting that latent viral reservoirs within gingival connective tissue might silently predispose to future periodontal breakdown during episodes of immune fluctuation or local microbial imbalances [18]. Also, latent HSV-I and II, in the context of compromised cytotoxic immune surveillance, may similarly periodically reactivate, potentially acting as pro-inflammatory catalysts that intensify the local immune response. These episodic viral flare-ups might intensify the inflammatory burden within the periodontal tissues, especially in an environment already burdened by microbial imbalances and chronic immune dysregulation, potentially contributing to the transition from gingivitis to more severe forms such as necrotizing gingivitis and periodontitis.

Fungal profiles were not investigated among pediatric subjects with HIV; however, *Candida* species are frequently isolated in HIV-positive adult individuals, even in the absence of overt mucosal lesions [186]. HIV-related depletion of Th17 cells and the disruption of the IL-17/IL-22 axis may impair antifungal defenses, promoting opportunistic fungal colonization that might contribute further to polymicrobial dysbiosis and heighten vulnerability to periodontal disease.

Moreover, the chronic inflammatory pathway generated by persistent viral infection and microbial colonization can reciprocally influence systemic immune dysfunction, in which periodontal disease may amplify HIV-associated immune activation and systemic inflammation [141]. This interplay is particularly evident in necrotizing periodontal diseases, which could be common in HIV-positive patients, reflecting an opportunistic infection dynamic potentiated by profound immune deficits [185,187,188].

Moreover, analysis of GCF in HIV-positive individuals showed distinctive cytokine signatures that may further explain the mechanisms linking systemic immunodeficiency to periodontal diseases. For instance, GCF in HIV-positive adults with chronic periodontitis displayed elevated levels of INF-α, whose elevated concentrations were found to be associated with increased probing depth and attachment loss, suggesting that antiviral immune activation may coincide with periodontal tissue breakdown [189]. In perinatally HIV-infected youth with gingivitis, GCF profiles showed increased 1β, TNF-α, IFN-γ, and MMP-9, suggesting that mucosal inflammation can remain active even in early stages of periodontal disease and may reflect broader systemic immune activation or predispose individuals to developing periodontal disease [190].

In pediatric individuals with malnutrition/kwashiorkor, the related microbial profile was reported in necrotizing gingivitis [104]. Although direct bacterial profiling was limited among these subjects [104], necrotizing gingivitis has been widely associated with Spirochetes and Gram-negative rods, such as *Bacteroides* species [191]. Indeed, chronic malnutrition affects the hypothalamic–pituitary–adrenal axis, leading to persistent hypercortisolism, which impairs neutrophil recruitment, suppresses phagocytosis, and weakens chemotaxis [192]. It is conceivable that such endocrine alterations not only suppress immune surveillance but also serve as metabolic substrates, thereby enhancing the motility and colonization of periodontal pathogens, which may facilitate the establishment of pathogenic biofilms. For instance, elevated cortisol levels have been shown to enhance the growth of *Fusobacterium nucleatum* and upregulate genes involved in protease production and iron acquisition, which are critical factors contributing to tissue destruction and disease progression [154,193]. Furthermore, chronic cortisol elevation may support the proliferation of other anaerobic Gram-negative species, including *Porphyromonas gingivalis* and *Bacteroides* spp., which are involved in necrotizing gingivitis as well as more advanced forms of periodontal disease, such as chronic periodontitis [194,195].

Additionally, micronutrient deficiencies typically observed in malnutrition, such as zinc, vitamin A, and B-complex vitamin deficiencies, may compromise epithelial barrier integrity and mucosal repair mechanisms [192], further amplifying susceptibility to microbial invasion. Sarcopenia and hypoalbuminemia, common in severely malnourished children [192], may also impair the synthesis of immunoglobulins and antimicrobial peptides, exacerbating host vulnerability.

The reported viral profile in this pediatric population [104] revealed the presence of CMV, EBV, HSV, and HHV-6 within necrotizing gingivitis lesions, whereas HPV and parvovirus B19 were absent. It could be plausible that malnutrition-induced T-cell dysfunction and chronic systemic inflammation promote herpesvirus reactivation [192]. The co-presence of multiple herpesviruses may reflect a complex interplay whereby viral reactivation may have reinforced immune dysregulation and microbial imbalance.

Though fungal colonization was underexplored, candidal overgrowth might be likely, given impaired salivary IgA production, epithelial atrophy, and disrupted iron homeostasis characteristic of malnutrition [83,191,196].

Intriguingly, despite this profound immunological vulnerability, a subset of malnourished children harboring latent herpesviruses have been reported to maintain a clinically healthy periodontium [12]. This paradox may suggest that systemic nutrient depletion may impose selective ecological constraints on the oral microbiome, potentially limiting the colonization or overgrowth of highly virulent species through mechanisms such as nutrient competition, residual immune regulation, or microbial antagonism within the biofilm community. In this light, kwashiorkor may not only predispose to necrotizing periodontal conditions but, in certain contexts, also paradoxically suppress the microbial configurations required to initiate overt disease.

### 9.3. Multisystem Genetic Syndromes

The reported microbial profiles in children with Down syndrome with periodontitis included a broad spectrum of periodontal pathogens. Among these, *Porphyromonas gingivalis*, *Treponema denticola*, *Tannerella forsythia*, and *A. a.* have been identified in Down syndrome-associated periodontitis, along with *Filifactor alocis*, *Fretibacterium* spp., *Peptostreptococcus* spp., and *Desulfobulbus* spp. [197,198]. Notably, these microbial profiles resemble those reported in adult populations with Down syndrome, suggesting a possible early establishment and persistence of dysbiotic communities across the lifespan [199]. It may be conceivable that this microbial landscape is shaped not by age but by the immunological and anatomical features characteristic of Down syndrome.

Moreover, the reported full-mouth plaque scores in children with periodontitis and Down syndrome [18,24] reached 96.67±6.83%, underscoring a profound plaque accumulation, which may provide ideal conditions for pathogenic biofilm maturation and persistence when coupled with compromised host defenses. Radiographic findings from the same pediatric cohort revealed alveolar bone loss in all six subjects with periodontitis, including one case with destruction exceeding half of the root length, further reinforcing the aggressive disease pattern [24].

Additionally, reduced salivary flow and altered composition of antimicrobial peptides in DS saliva, such as lower β-defensin and cathelicidin levels, may provide selective advantages to aciduric and proteolytic bacteria [197]. It is plausible that anatomical anomalies common in these syndromes—macroglossia, high palatal vault, hypotonia, and anterior open bite—not only impair mechanical self-cleansing but may also create protected subgingival microenvironments with low oxygen tension and reduced shear stress, thus facilitating the proliferation of anaerobic species such as *Fusobacterium nucleatum* and *Treponema* spp. [198].

From a systemic standpoint, Down syndrome is associated with dysregulated innate immunity—particularly, impaired neutrophil chemotaxis, oxidative burst, and phagocytosis—which may diminish the host’s ability to contain microbial overgrowth and resolve periodontal inflammation [200]. It is plausible that such defects allow for early and persistent colonization by virulent taxa, even in the absence of clinical signs. Subgingival profiles suggest that even Down syndrome individuals with an apparently healthy periodontium may harbor pathogens typically associated with periodontitis [18,24,200,201].

Indeed, viral findings in this population further complicate general health and periodontal status-related considerations. EBV, CMV, and HSV were detected in children with Down syndrome with periodontitis, including one case of viral co-infections [24]. These viruses may establish latent reservoirs and periodically reactivate in the setting of immune dysregulation, exacerbating local inflammation and interfering with antigen-specific responses [13,136,201].

Although fungal profiling was not reported among a pediatric cohort, the frequent detection of *Candida albicans* in Down syndrome individuals—particularly under conditions of antibiotic use, xerostomia, or nutritional deficits—may indicate an additional layer of complexity within the periodontal ecosystem [200,201].

### 9.4. Metabolic and Hematologic Disorders Affecting the Immune System

In children with Fanconi anemia and diabetes mellitus with periodontitis, the reported microbial content included *Campylobacter* species, *Fusobacterium* species, *Parvimonas micra*, *A. a.*, *Fusobacterium*, and *Parvimonas micra*, alongside viral agents like CMV and HSV, and the fungal species *Candida albicans* [170].

In Fanconi anemia, the hallmark of genomic instability and defective DNA repair mechanisms contributes to bone marrow failure and impaired leukocyte function [162,170]. It may be plausible that the systemic oxidative stress characteristic of this condition alters redox signaling within the periodontal niche, thereby supporting periodontal pathogen bacteria such as *Fusobacterium* and *Parvimonas* species [202].

Moreover, impaired neutrophil recruitment and reduced levels of salivary antimicrobial peptides may weaken the first line of mucosal defense, allowing colonization by leukotoxin-producing species such as *A. actinomycetemcomitans*, which may further exacerbate epithelial damage and trigger severe periodontal inflammation [202,203].

In parallel, diabetes mellitus promotes a hyperglycemic environment that not only impairs immune cell function but also provides metabolic substrates conducive to microbial overgrowth [106]. Elevated glucose levels in saliva and gingival crevicular fluid may enhance fungal adhesion, hyphal transformation, and biofilm maturation of *Candida albicans*, a species often isolated in diabetic individuals with periodontitis [204,205].

Moreover, the detection of members of the *Herpesviridae* family [170] may exploit the impaired T-cell-mediated immunity observed in both conditions to establish latency and reactivate intermittently [174,206].

## 10. Discussion

The present narrative review aimed to explore the periodontal status (ranging from healthy periodontium, gingivitis, periodontitis, and necrotizing gingivitis/periodontitis) and the related subgingival and/or salivary microbial profiles of pediatric subjects (≤18 years of age), focusing also on the state of health or systemic diseases affecting the pediatric population.

An opposing symmetry in periodontal disease distribution between systemically healthy and compromised children emerged [18,21], suggesting that systemic health status influences not only the presence of periodontal disease but also its presentation, severity, and clinical distribution.

Indeed, in cohorts of systemically healthy children, the majority of individuals investigated exhibited a clinically healthy periodontium; conversely, in systemically compromised pediatric subjects, periodontal disease, particularly in its severe forms, was markedly more prevalent [18,21]. Notably, among systemically healthy individuals with periodontitis, the disease manifested more often with localized patterns, particularly molar–incisor [21]. In contrast, systemically compromised subjects were more likely to present with generalized forms, including necrotizing forms that were absent in systemically healthy children [18].

In addition to that, the reported clinical periodontal parameters also provide valuable insights into the differences across systemically healthy and compromised pediatric populations, as generally deeper PPD, greater BoP, and CAL were reported in the systemically compromised cohort [18], likely influenced by impaired immune responses and increased susceptibility to microbial invasion and inflammation due to the underlying medical conditions.

### 10.1. Which Microbial Shifts Herald Progression from Health to Gingivitis?

Despite differing systemic conditions, recent evidence [18,21] suggests that in the context of periodontal disease, both systemically healthy and compromised pediatric subjects consistently harbor key pathogens belonging to the red and orange complexes, including *Porphyromonas gingivalis*, *Tannerella forsythia*, *Prevotella intermedia/nigriscens*, and *Fusobacterium nucleatum*. This recurrence highlights that some bacterial species may represent a microbial signature of periodontal disease, transcending the underlying systemic status and acting as fundamental contributors to disease onset or progression.

However, while this microbial consistency underscored shared pathogenicity mechanisms, a difference was found concerning ecological complexity [18,21]. Indeed, in systemically compromised individuals, the bacterial profile appeared less diverse and more constrained [18], potentially reflecting the impact of an impaired immune system on the regulation of oral microbiota. The lowered microbial heterogeneity may indicate a weakened ability to sustain ecological balance and immunological control, fostering selective colonization by specific species. Conversely, systemically healthy pediatric subjects tended to exhibit a more variegated and complex bacterial profile [21], likely supported by the proper immune system regulation and robust host–microbiome interactions.

Moreover, children affected by systemic diseases may represent permissive hosts for certain bacterial species, some of which are associated with systemic complications and are only sporadically encountered in systemically healthy counterparts [88,172].

It might be plausible that the reduced immunological surveillance in these individuals facilitates the persistence of limited microbial taxa that would otherwise be competitively excluded in a systemically healthy status, thereby enriching the biofilm with limited but more virulent bacteria, altering microbial ecology, and predisposing to more severe or advanced forms of periodontal disease.

### 10.2. How Does the Pediatric Bacterial Periodontal Profile Compare to the Adult One?

The composition of the subgingival microbiota in pediatric populations exhibited both similarities and differences when compared to that of adults across different periodontal conditions. Indeed, comparing these profiles with those of adults might provide valuable insights when interpreting the microbial signatures currently observed in children.

In clinically healthy periodontium, adults exhibited a low-diversity, health-associated microbiota characterized by Gram-positive and saccharolytic species, including *Streptococcus oralis*, *Actinomyces naeslundii*, and *Actinomyces gerencseriae*, consistent with biofilm stability, inhibition of pathogenic overgrowth, and immune tolerance [207]. Additional genera such as *Rothia* and *Veillonella* were also detected, supporting health-compatible metabolic interactions within the biofilm [208]. Notably, typical periodontal pathogens such as *Porphyromonas gingivalis*, *Tannerella forsythia*, *Campylobacter rectus*, *Prevotella intermedia*, and *Fusobacterium nucleatum* were either absent or present only in minimal amounts, as reported by low relative abundances in quantitative assessments [207].

Similarly, in the present narrative review, pediatric subjects with a healthy periodontium were characterized by early-colonizing, saccharolytic taxa, including *Streptococcus*, *Actinomyces*, *Veillonella*, and *Capnocytophaga* species. In parallel with the adult profile, pathogenic species were minimally represented or present in non-pathogenic quantities, reflecting a less complex and less anaerobic microbial ecology. When detected, these pathogens were more frequently observed in pediatric subjects with underlying systemic diseases, suggesting that systemic conditions may influence the presence of potentially pathogenic bacteria in periodontal health in this population.

In gingivitis, the adult microbiota exhibited increased diversity and enrichment of anaerobic and proteolytic species, including *Fusobacterium nucleatum*, *Prevotella intermedia*, *Campylobacter gracilis*, *Campylobacter rectus*, *Selenomonas* spp., and *Eikenella corrodens* [207,208,209]. Concurrently, facultative pathogens such as *Klebsiella* spp. and *Escherichia* spp. have also been identified in adult gingivitis, particularly in association with greater inflammatory levels and poor oral hygiene status [209].

Similarly, pediatric gingivitis was characterized by species including Prevotella intermedia, Fusobacterium nucleatum, Campylobacter rectus, Peptostreptococcus micros, and Capnocytophaga spp. However, in children, increased levels of Prevotella intermedia and Fusobacterium nucleatum were associated with pronounced marginal erythema and BoP. Unlike adults, Klebsiella spp. and Escherichia spp. were not found to be associated with the inflammatory burden of gingivitis in pediatric subjects. Notably, Selenomonas spp., which are reported as transitional species in adult gingivitis, were less frequently detected in children, suggesting a reduced shift in microbial diversity in the pediatric condition.

In periodontitis, the adult biofilm was characterized by an enriched microbial species, including key pathogens such as *Porphyromonas gingivalis*, *Tannerella forsythia*, and *Treponema denticola* [207,208]. Additional periodontal pathogens frequently reported in adults included *Fusobacterium nucleatum*, *Parvimonas micra*, *Prevotella* spp., and *A. a.* [209].

In pediatric periodontitis, some of these pathogens were also reported, including *Porphyromonas gingivalis*, *Fusobacterium nucleatum*, *Capnocytophaga* spp., and *A. a*. However, the classic red complex bacteria tended to be inconsistently present and usually at lower relative abundances compared to adults. Notably, *A.a* was more frequently found in localized aggressive forms of periodontitis in children, especially those with underlying systemic conditions. Unlike adults, *Prevotella* spp. were detected in fewer cases and associated mainly with genetic syndromes such as Papillon–Lefèvre and Kostmann syndromes.

Although similarities were observed between adult and pediatric populations in the overall transition from a symbiotic to a dysbiotic microbial community during periodontal disease progression, distinct differences in microbial composition and complexity were also noted. In pediatric subjects, the transition is characterized by a less complex microbial profile, potentially ascribable to differences in host immune responsiveness, microbial colonization patterns, and environmental, dietary, and hygiene-related factors.

### 10.3. What Roles Do Viruses Play on Pediatric Periodontal Status?

In systemically healthy pediatric individuals, viruses, in particular, HHVs, including EBV, CMV, and HSV, were more frequently detected in periodontitis and sporadically in gingivitis, suggesting a potential viral involvement primarily in chronic inflammatory conditions [21]. This distribution pattern may suggest that in immunocompetent hosts, viral reactivation may be favored by prolonged exposure to a dysbiotic biofilm and sustained local inflammation, rather than by transient or mild gingival changes. In this environment, *Herpesviridae* could contribute to the progression and severity of periodontal destruction, modulating host immune responses and amplifying tissue-destructive pathways [40,57,59,141].

Conversely, in children with systemic conditions, a different trend emerged, as CMV and HSV were predominantly identified in acute periodontal diseases, particularly necrotizing gingivitis [18]. These findings may reflect the underlying systemic conditions, in which the impaired systemic immunity fails to contain viral replication even in the early phases of periodontal breakdown, facilitating rapid tissue destruction.

Among pediatric subjects with a clinically healthy periodontium, CMV was identified in both systemically healthy and compromised subjects [18,21], potentially suggesting that its presence may not be strictly dependent on systemic immune status. Instead, its persistence could be related to local immune surveillance or mechanisms of viral latency that might be active regardless of broader systemic conditions.

EBV, by contrast, was largely absent in both healthy periodontium cohorts [18,21], indicating that, despite the known affinity for the sulcular and junctional epithelium regions, which represent potential entry portals and latent viral reservoirs [210], its pathogenic expression may require additional enabling factors, such as the presence of specific supporting bacteria species. Interestingly, the lack of *A. a.* observed may partially explain the limited reactivation of EBV, as this bacterium has been implicated in viral activation and host immune evasion mechanisms [151,211]. EBV-1 appeared more frequently in systemically healthy individuals than in those with systemic compromise [18,21], possibly reflecting more efficient viral persistence mechanisms in the presence of intact immune regulation. Conversely, systemic impairments may hinder viral replication and the microbial and immunological conditions required to sustain such latency.

Additionally, no viral co-infection was reported in healthy periodontal status [18,21], suggesting that a stable microbial environment associated with periodontal health may not support the simultaneous persistence of multiple herpesviruses. Although crevicular parameters were not directly examined, one could hypothesize that components of gingival crevicular fluid, such as antimicrobial peptides and cytokines, may have actively contributed to viral clearance [79,212,213]. Furthermore, the distinct metabolic needs and replication strategies of various herpesviruses may inherently limit their coexistence, as competition for cellular resources could preclude simultaneous persistence [136].

### 10.4. What Roles Do Viruses and Fungi Play on Pediatric Periodontal Status?

The contribution of fungi to the pathogenesis of periodontal disease in the pediatric population remains underexplored [18,21]. Although their role is still not established, emerging evidence [18,21] suggests that fungal species such as *Candida albicans* and *Candida dubliniensis* may occasionally be present under specific host and environmental conditions. Their detection was reported more in systemically compromised children, where immune dysfunction or metabolic dysregulation may create permissive conditions for fungal colonization and transition from commensalism to pathogenicity [18,21]. Indeed, in systemically healthy pediatric subjects, fungal presence within periodontal tissues was rarely reported and often absent, possibly reflecting an intact mucosal immunity and balanced microbial ecology that prevents fungal overgrowth [18,21]. However, in altered contexts, such as in periodontitis, especially with chronic inflammation, fungi may find a favorable niche, potentially contributing to disease progression through hyphal penetration, epithelial disruption, and the release of virulence factors [38,103].

Conversely, in systemically compromised pediatric patients, the impaired immune surveillance, hematopoietic deficiencies, or hyperglycemic conditions could not only facilitate fungal adhesion and biofilm formation but also intensify the inflammatory cascade through mechanisms involving oxidative stress and advanced glycation end-products, potentially causing alterations that may provide a biologically plausible context for *Candida* spp. to act not merely as opportunistic colonizers but also as potential contributors to periodontal destruction.

These findings suggest the complex interplay between bacteria, viruses, and fungi, influencing the onset, progression, and clinical presentation of periodontal disease in pediatric subjects, which is finely modulated by the host’s immune competence and the presence of systemic conditions.

### 10.5. Study Limits and Future Perspectives

This article represents a narrative review rather than a systematic one, and as such, it does not include a formal quality assessment of the included studies nor a meta-analysis. In addition, among the main limitations of the available literature is the considerable heterogeneity in methodologies used across studies. This includes substantial variation in sample size, with several investigations based on case reports or small cohorts, which limits the statistical power and generalizability of findings [127,130].

Additionally, the use of different microbial detection techniques, such as culture-based methods versus DNA-based techniques (e.g., PCR, nested-PCR, real-time PCR), introduces variability in sensitivity and specificity, potentially leading to under- or over-estimation of microbial presence. For instance, while culture methods can miss non-cultivable species, PCR-based approaches may detect non-viable organisms, impacting clinical interpretation [10,170].

Differences in sampling strategies (e.g., saliva vs. subgingival plaque, pooled vs. site-specific samples), targeted microbial genes, and timing relative to periodontal therapy further complicate cross-study comparisons [28,43]. Moreover, few studies reported standardized criteria for periodontal diagnosis or consistent clinical parameters (e.g., probing depth, clinical attachment level), limiting reproducibility.

Another important consideration is the geographic and racial variability in pediatric populations. Many studies were conducted in highly specific demographic settings—such as Nigeria [104], Brazil [125], and Lebanon [170]—often without accounting for ethnic, socioeconomic, or environmental confounders, which could influence both the microbiome composition and host immune response [214]. This underlines the need for multicenter studies including ethnically and geographically diverse cohorts.

It is also important to emphasize the limited and largely indirect evidence available regarding viral and fungal involvement in pediatric periodontitis. Much of the current understanding in this area is extrapolated from adult studies or based on small-scale pediatric investigations, making it difficult to draw definitive conclusions. As a result, many of the interpretations presented herein remain speculative, particularly in the absence of longitudinal data or pediatric-specific experimental models.

Future studies should focus on elucidating the integrated roles of bacteria, viruses, and fungi in systemically healthy and compromised pediatric individuals. Particular attention should be directed toward identifying specific microbial patterns and interkingdom interactions that correlate with disease onset, severity, and distribution. Moreover, the adoption of newly available high-resolution technologies for microbial and host profiling may be essential in elucidating the functional networks underlying periodontal tissue breakdown in children. By capturing the microbial profile complexity and the related interactions with the immune system, these tools could enhance the ability to understand pathogenic mechanisms, stratify risk, and develop personalized preventive and therapeutic approaches in pediatric periodontology.

## 11. Conclusions

Periodontal health and disease in pediatric subjects result from a dynamic interplay between the oral microbiota, comprising bacteria, viruses—in particular, *Herpesviridae*—and fungi, the host’s immune system, and systemic health or disorders.

While most existing research focuses on periodontal health and disease in adult subjects, beyond the well-established bacterial role, increasing evidence underscores the involvement—in particular, of HHVs—in the pathogenesis of periodontal diseases in pediatric subjects, both those who are systemically healthy and those affected by systemic conditions.

Polymicrobial inter-kingdom synergy appears to play a central role in the transition from a eubiotic to a dysbiotic oral state.

Pediatric subjects affected by systemic disorders (e.g., genetic syndromes, immunodeficiencies, malnutrition) exhibit a higher susceptibility to more severe and rapidly progressive periodontal disease forms.

Nevertheless, the lack of pediatric longitudinal studies investigating periodontal disease progression after periodontal treatment and the related changes in microbiological composition has limited the understanding and exploration of the oral viriome and mycobiome over time.

Understanding the periodontal microbial profile in pediatric subjects may offer critical insights to improve the clinical management of periodontal diseases in children, as well as to develop prevention strategies, particularly in systemically compromised pediatric subjects, who are at higher risk of being affected by aggressive and rapidly progressive periodontal diseases, and microbiome-centered health promotion during childhood.

## Figures and Tables

**Table 1:** Predominant periodontal bacterial pathogens: key microbiological characteristics and role in periodontal health and diseases.

Bacterium	Socransky Complex	Microbiological Features	Main Associated Periodontal Conditions	Key Periodontal Role
*Streptococcus* spp. (*S. oralis*, *S. mitis*, *S. sanguis*) [42,43]	Yellow	Gram-positive cocci Facultative anaerobes	Periodontal health	Early colonizers Maintaining oral eubiosis
*Actinomyces* spp. (*A. naeslundii*, *A. oris*) [42,43]	Blue	Gram-positive rods Facultative anaerobes/anaerobic	Periodontal health	Early colonizers Maintaining oral eubiosis
*Veillonella parvula* [44]	Purple	Gram-negative coccus Anaerobic	Periodontal health	Early colonizers
*Capnocytophaga* spp. [45]	Green	Gram-negative rods Facultative anaerobes/microaerophiles	Gingivitis Periodontal health	Early colonizers
*Fusobacterium nucleatum* [46,47,48]	Orange	Gram-negative rod Anaerobic	Gingivitis Periodontitis Necrotizing forms	Bridge bacterium, which facilitates co-aggregation
*Prevotella intermedia* [46,47,48]	Orange	Gram-negative rod Microaerophiles	Gingivitis Periodontitis Necrotizing forms	Involved in inflammatory response and biofilm accumulation
*Campylobacter rectus* [29]	Orange	Gram-negative rod, motile Anaerobic	Gingivits Periodontitis	Involved in inflammatory response and tissue invasion
*Parvimonas micra* [29]	Orange	Gram-positive coccus Anaerobic	Gingivits Periodontitis	Enhances inflammation
*Porphyromonas gingivalis* [49]	Red	Gram-negative rod Anaerobic	Gingivitis Periodontitis	Evades immune response and disrupts host–microbiome balance
*Tannerella forsythia* [21]	Red	Gram-negative rod Anaerobic	Gingivitis Periodontitis	Produces proteolytic enzymes and surface-layer proteins that promote epithelial invasion and immune evasion
*Treponema denticola* [50,51]	Red	Gram-negative spirochete, motile Anaerobic	Gingivitis Periodontitis Necrotizing forms	Highly proteolytic spirochete; invades tissues and promotes necrotizing forms
*Aggregatibacter actinomycetemcomitans* [52,53,54]	None	Gram-negative coccobacillus Facultative anaerobic	Aggressive periodontitis	Major pathogen in aggressive periodontitis; JP2 clone produces leukotoxin and impairs immune defense in adolescents (12–19 years old)

**Table 2:** Inter-kingdom and viral co-infection mechanisms in periodontal disease.

Microorganisms Species	Periodontal Diseases	Interactions
*Porphyromonas gingivalis*, *Fusobacterium nucleatum*, and EBV-I, EBV-II, CMV	Generalized periodontitis MIPP	*Porphyromonas gingivalis* and *Fusobacterium nucleatum* trigger viral reactivation, exacerbating the inflammatory status by the relapse of metabolites like butyric acid [70].
*Porphyromonas gingivalis* and *Candida albicans*	Periodontitis	*Candida albicans* promoted *Porphyromonas gingivalis* growth and virulence, creating an anaerobic environment and promoting bacterial invasion and toxin release. *Porphyromonas gingivalis* favors *Candida albicans* adhesion [67,69].
*A.actinomycetemcomitans*, *Fusobacterium nucleatum*, and *Candida albicans/dubliniensis*	Periodontitis	*Fusobacterium nucleatum* and *A. actinomycetemcomitans* promoted co-aggregation and inhibited *Candida* filamentation and growth, releasing quorum-sensing molecules as autoinducer-2 [67,69,71,72].
*A.actinomycetemcomitans* and CMV	Periodontitis Necrotizing gingivitis	Active CMV infection enhanced the initial bacterial colonization, destroying the epithelial cells of the periodontal pocket and exposing new attachment receptors for bacteria, thus enhancing adherence and growth in a dose-dependent manner [13].
*Porphyromonas gingivalis* and HHVs	Periodontitis	HHV infections impair neutrophil, macrophage, and complement functions, reducing antibody-mediated bacterial clearance and favoring the overgrowth of *Porphyromonas gingivalis*; the bacteria, conversely, release proteases as gingipains, degrade antiviral cytokines (IL-6 and IL-8), and contribute to the reactivation of latent herpesviruses, further amplifying inflammation and tissue destruction [13].
EBV and/or CMV and/or HSV	Active periodontitis Acute necrotizing gingivitis	The co-infection of EBV and CMV enhanced the cytotoxic T-cell responses and the release of pro-inflammatory cytokines. Furthermore, the co-infection by two HHVs leads to a reciprocal transactivation of the latent viral genomes and the viral reactivation [13].
HPV or human parvovirus B-19 and HHVs	Necrotizing gingivitis Periodontitis	A putative negative competitive interaction suggested that HHVs inhibit replication of non-herpesvirus such as HPV and human parvovirus B-19, modulating immune system responses. In fact, in both necrotizing gingivitis and periodontitis, HPV and human parvovirus B-19 were not detected in positive HHV samples in pediatric subjects [18].

Acronyms: HHVs, “human herpesviruses”; CMV, “cytomegalovirus”; EBV, “Epstein–Barr virus”; HSV, “human simplex virus”; HPV, “human papillomavirus.”

## Data Availability

No new data were created or analyzed in this study. Data sharing is not applicable to this article.
